# Granulocyte colony‐stimulating factor is not pathogenic in lupus nephritis

**DOI:** 10.1002/iid3.430

**Published:** 2021-05-07

**Authors:** Timothy A. Gottschalk, Fabien B. Vincent, Alberta Y. Hoi, Margaret L. Hibbs

**Affiliations:** ^1^ Leukocyte Signalling Laboratory, Department of Immunology and Pathology, Central Clinical School Monash University Melbourne Victoria Australia; ^2^ Centre for Inflammatory Diseases, School of Clinical Sciences at Monash Health Monash University Clayton Victoria Australia

**Keywords:** autoimmune disease, granulocyte colony‐stimulating factor, inflammation, Lyn tyrosine kinase, systemic lupus erythematosus

## Abstract

Systemic lupus erythematosus (lupus) is an autoimmune disease characterized by autoantibodies that form immune complexes with self‐antigens, which deposit in various tissues, leading to inflammation and disease. The etiology of disease is complex and still not completely elucidated. Dysregulated inflammation is an important disease feature, and the mainstay of lupus treatment still utilizes nonspecific anti‐inflammatory drugs. Granulocyte colony‐stimulating factor (G‐CSF) is a growth, survival, and activation factor for neutrophils and a mobilizer of hematopoietic stem cells, both of which underlie inflammatory responses in lupus. To determine whether G‐CSF has a causal role in lupus, we genetically deleted G‐CSF from Lyn‐deficient mice, an experimental model of lupus nephritis. Lyn^−/−^G‐CSF^−/−^ mice displayed many of the inflammatory features of Lyn‐deficient mice; however, they had reduced bone marrow and tissue neutrophils, consistent with G‐CSF's role in neutrophil development. Unexpectedly, in comparison to aged Lyn‐deficient mice, matched Lyn^−/−^G‐CSF^−^^/−^ mice maintained neutrophil hyperactivation and exhibited exacerbated numbers of effector memory T cells, augmented autoantibody titers, and worsened lupus nephritis. In humans, serum G‐CSF levels were not elevated in patients with lupus or with active renal disease. Thus, these studies suggest that G‐CSF is not pathogenic in lupus, and therefore G‐CSF blockade is an unsuitable therapeutic avenue.

## Graphical abstract

Lupus nephritis is perpetuated by unrestrained inflammation, potentially driven by the hematopoietic growth factor granulocyte colony‐stimulating factor (G‐CSF). Lupus‐prone Lyn‐deficient mice lacking G‐CSF were not protected from the development of autoimmunity and, in fact, exhibited exacerbated indices of kidney pathology. Furthermore, G‐CSF levels in human serum were not elevated in systemic lupus erythematosus patients or in correlation with renal disease, overall indicating that G‐CSF is not a major pathogenic factor in lupus nephritis.

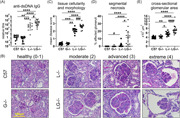

**Abbreviations**
BM: bone marrow
DCs: dendritic cells
G‐CSF: granulocyte colony‐stimulating factor
HC: healthy control
SLE: systemic lupus erythematosus

## 1 INTRODUCTION

Systemic lupus erythematosus (SLE, lupus) is a biologically and clinically heterogeneous, multisystem autoimmune disease characterized by the production of antiself‐autoantibodies, primarily directed against nuclear components.^1^ These self‐reactive antibodies can form immune complexes with self‐antigens that can deposit in tissues and invoke an inflammatory response, which involves cytokine and chemokine release, promoting inflammatory cell recruitment and activation, ultimately leading to tissue damage. Renal involvement in the form of lupus nephritis is common and contributes significantly toward the disease morbidity and mortality. Although loss of B cell tolerance leading to B lymphocyte activation and the production of autoantibodies triggers the onset of lupus, other factors are also involved in the disease process, including dysregulation of innate and other adaptive immune responses. Thus, therapies targeting both arms of the immune system may be important for controlling lupus.^2^, ^3^

Granulocyte colony‐stimulating factor (G‐CSF), which is produced by endothelium and stromal cells in response to inflammatory stimuli,^4^ promotes neutrophil production from the bone marrow (BM) and supports neutrophil survival, activation, and function. While G‐CSF is important in innate immune responses, recent studies have shown that excess G‐CSF can be pathogenic in diverse autoimmune and inflammatory diseases such as inflammatory arthritis,^5^ experimental multiple sclerosis,^6^ and chronic obstructive pulmonary disease.^7^ Although the role of G‐CSF in SLE is still unclear, it is implicated in a number of pathogenic processes commonly seen in lupus including expansion and activation of neutrophils, mobilization of BM stem cells, and expansion of myeloid effector cells via extramedullary hematopoiesis, activation and maturation of dendritic cells (DCs), and skewing of T cell responses. In many cases, neutrophils are expanded in SLE and neutrophil gene signatures are frequently found in patients with active disease,^8^, ^9^, ^10^, ^11^ which further implicates G‐CSF as a potential pathogenic factor in lupus.

Lyn‐deficient mice represent an experimental model of lupus,^12^, ^13^ which spontaneously develop disease resembling SLE.^14^ Inflammation underlies disease development, as shown by deletion of the proinflammatory cytokine interleukin (IL)‐6, which prevented lupus nephritis, while the mice remained autoimmune‐prone.^15^, ^16^ G‐CSF levels have been found to be elevated in the serum of aged Lyn^−/−^ mice in settings where the mice were engineered to show augmented tissue inflammation and disease such as in the context of IL‐10 deficiency^17^ or when Lyn was deleted specifically from DCs.^18^ Lyn^−/−^ mice show an expansion of neutrophils during lupus progression,^15^, ^19^ and the neutrophils are hyperactive,^20^ implying a role in disease. G‐CSF also stimulates hematopoietic stem cell production and mobilization into circulation,^21^ which occurs during disease development in Lyn‐deficient mice,^22^, ^23^ suggesting that this phenotype may be driven by elevated G‐CSF. Altogether these studies suggest that G‐CSF may play a pathogenic role in exacerbating inflammation in this model. Therefore, we generated Lyn‐deficient mice lacking G‐CSF to determine if we could moderate development of lupus by preventing myeloid cell‐dependent inflammation.

## 2 MATERIALS AND METHODS

### 2.1 Animals

C57BL/6 background Lyn^−^^/−^ (L^−/−^) mice^12^, ^15^ and G‐CSF^−/−^ (G^−/−^) mice^7^, ^24^ have been described. Lyn^−/−^G‐CSF^−^^/−^ (LG^−/−^) mice were generated by intercrossing and genotyping, and were ostensibly healthy and fertile. C57BL/6 (C57) controls were from Monash Animal Services SPF facility (Clayton, Australia), where all mice were housed from weaning. Mice were analyzed at 36 weeks of age, when autoimmune kidney pathology is severe in Lyn^−/−^ mice.^15^ Research was conducted under the Australian Code for the Care and Use of Animals for Scientific Purposes, and approval was obtained from the Alfred Research Alliance Animal Ethics Committee (E/1688/2016/M).

### 2.2 Quantification of G‐CSF in mouse sera

Serum from aged mice was analyzed by custom Bio‐Plex Pro Assay Kit (Bio‐Rad) as per the manufacturer's instructions. Results were revealed using Bio‐Plex 200 System with the Bio‐Plex Manager 4 software.

### 2.3 RT‐PCR

RNA from lung tissue was obtained by Trizol/Chloroform extraction and converted to complementary DNA (cDNA) using the FireScript RT kit (Solis Biodyne) as per manufacturer's instructions. RT‐PCR used validated primers for *Csf3* ^45^ and *18S* ^46^ with PowerUp SYBR Green Master Mix (Applied Biosystems) as per manufacturer's instructions for 40 cycles under single‐plex conditions (QuantStudio 7; Life Technologies). RT‐negative and template‐negative controls were included to validate cDNA‐specific amplification. Samples were run in triplicate and cycle threshold values were determined by automatic threshold analysis (QuantStudio Software), from which the triplicate average was used to determine relative gene expression using the 2^−ΔΔCt^ method, with *18S* as the housekeeping control.

### 2.4 Flow cytometry

BM cells were flushed from the femurs and single‐cell suspensions of spleen were prepared by extrusion. Red blood cells were lysed with ACK lysis buffer, Fcγ receptors were blocked using Fc Block (clone 2.4G2), and fluorophore‐conjugated monoclonal antibodies were used to stain cells. Labeled cells were analyzed by flow cytometry using an LSRFortessa X‐20 Instrument (BD Biosciences). Viable cells were gated by fluorogold exclusion and single cells were further assessed as follows: erythrocytes: Ter119^+^CD71^±^; BM neutrophils: Lineage(B220+NK1.1+Thy1.2+CD115+)^−^c‐Kit^−^SiglecF^−^CD11b^+^Ly6G^+^; splenic neutrophils: CD11b^+^CD115^−^SiglecF^−^Ly6G^+^; monocytes: CD11b^+^CD115^+^, further subdivided as classical (Ly6C^+^CD43^−^) or nonclassical (Ly6C^−^CD43^+^); B cells: B220^+^CD19^+^, further subdivided as follicular (CD21/35^lo^CD23^+^), marginal zone (CD21/35^hi^CD23^−^) or transitional (CD21/35^−^CD23^−^); plasma cells: B220^lo^CD138^hi^; CD4^+^ T cells: CD3ε^+^CD4^+^; CD8^+^ T cells: CD3ε^+^CD8α^+^. CD4+ and CD8^+^ T cells were further subdivided as effector memory (CD44^+^CD62L^−^). Total cell numbers were derived from cell counts after red blood cell lysis and cell proportions were classified by flow cytometry. The expression of cell surface activation markers was determined by normalizing their geometric mean fluorescence intensity (gMFI) on gated cell populations by dividing the gMFI of individual samples by the mean gMFI of the C57BL/6 group within each experiment, allowing data from multiple experiments to be pooled.

### 2.5 Detection of autoantibodies by enzyme‐linked immunosorbent assay

MaxiSorp 96‐well immunoplates (Nunc) were treated overnight with 50 µl/well of 50 µg/ml calf thymus DNA in phosphate‐buffered saline (PBS) (Sigma‐Aldrich). Coated plates were washed with PBS/0.5% Tween‐20, blocked with assay diluent (BD) for 60 min, and then serum samples were added. An in‐house high‐titer antinuclear antibody reference serum was used to generate a standard curve for derivation of arbitrary titer values. After incubation (2 h, room temp), plates were washed and goat anti‐mouse immunoglobulin G (H + L)‐horseradish peroxidase (Southern Biotech) detection antibody was added to the wells for a further 60 min. Colorimetric detection was achieved with TMB chromogenic substrates A and B (BD), and the reaction was stopped with 1.5M sulfuric acid. Plates were read on a MultiSkan GO microplate spectrophotometer (Thermo Fisher Scientific) at 450 nm with wavelength correction at 595 nm. Relative titers were established by plotting sample optical density values against the standard curve.

### 2.6 Kidney histology

Mouse kidneys were fixed in 10% neutral‐buffered formalin for 24 h, transferred to ethanol, and processed. Paraffin‐embedded sections of 3 µm were set on SuperFrost slides and stained with Periodic Acid–Schiff. Sections were imaged using an Olympus BX‐51 light microscope (Olympus Australia), taking more than eight images of the kidney cortex/sample, capturing at least 30 glomeruli per kidney. Polygon tool tracing with ImageJ software (NIH, 1.52d) was used to quantify the area of each glomerulus. A custom disease score describing the changes to glomerular cellularity (mesangial, endothelial and infiltrating) and morphology was derived based on the typical disease presentation of Lyn^−^^/−^ mice and classified as follows: 0: Normal glomerular cellularity and morphology; 1: Mild cellular expansion and/or early‐stage disrupted glomerular morphology that includes lobularity and/or Bowman's space enlargement; 2: Advanced cellular expansion with distinct disruption to glomerular morphology, pyknosis and/or karyorrhexis; 3: Severe cellular expansion/consolidation with advanced lobularity or fragmentation and/or enlargement of the Bowman's space with cellular infiltration (with or without crescent formation) and/or periglomerular cellular expansion; 4: End‐stage glomerular destruction, progressive or complete loss of cellularity, and/or distinct glomerular morphology. Segmental necrosis was scored as the proportion of glomeruli exhibiting necrotic lesions per kidney, as determined by diffuse or patches of anuclear pink staining. At least 30 glomeruli were analyzed per kidney for each parameter, with the mean value taken to represent each sample.

### 2.7 Human participants and clinical assessments

Patients aged 18 and over, fulfilling the 1997 American College of Rheumatology revised criteria or the Systemic Lupus International Collaborating Clinic criteria for SLE classification,^47^, ^48^ already enrolled in the Australian Lupus Registry & Biobank,^49^ were recruited for this study from the Monash Lupus Clinic (Clayton, Victoria, Australia) between June 2015 and September 2017. As part of the prospective collection of demographic and clinical data, disease activity was assessed using the hybrid SLE Disease Activity Index 2000 (SLEDAI‐2K).^50^ Renal‐specific disease activity was assessed using the four renal domain components of the SLEDAI‐2K (proteinuria, hematuria, pyuria, and urinary cast), as described^51^; an SLE patient was considered to have active renal disease if at least one of these four renal items of the SLEDAI‐2K was positive. HCs consisted of healthy subjects aged 18 and above who were recruited between February and August 2017 from Monash Medical Centre (Clayton, Victoria, Australia). All study participants gave written informed consent. This study was approved by the Monash Health Human Research Ethics Committee (13019A).

### 2.8 Quantification of G‐CSF from human sera

Quantification of serum G‐CSF concentration was conducted by Crux Biolabs (Bayswater, Victoria) using Quantibody® platform (Raybiotech), as part of a commercial 40‐plex panel (Human Inflammation Array 3: QAH‐INF‐3), as per the manufacturer's protocol. An arbitrary value of the upper limit of quantification (ULOQ) and half the lower limit of quantification (LLOQ) was assigned to serum G‐CSF values falling above and below the ULOQ and LLOQ, respectively.

### 2.9 Statistical analyses

For mouse studies, statistical significance was determined using Mann–Whitney nonparametric *U* test (for two groups) or Kruskal–Wallis *H* test, followed by Dunn's multiple comparisons test (for four groups), with horizontal bars on data representing the median ± interquartile range (IQR). Specific comparisons between C57BL/6 and G‐CSF^−/−^ groups and Lyn^−/−^ and Lyn^−/−^G‐CSF^−/−^ groups were also conducted using Mann–Whitney nonparametric *U* test. Horizontal bars represent median ± IQR where the data are displayed on a linear scale or geometric mean ± geometric *SD* where logarithmic data are displayed. Significance by Mann–Whitney test (represented by #) and Dunn's multiple comparisons test (represented by *) is denoted by *p* > .05 (not significant) not stated, not stated, *p* < .05 # or *, *p* < .01 ## or **, *p* < .001 ### or ***, *p* < .0001 #### or ****. Mouse data analyses utilized GraphPad Prism software (version 8.0.2). For human studies, serum G‐CSF concentrations were transformed into a binary variable as being either detectable or not detectable, using the LLOQ as a threshold. Comparison of proportions were assessed using Pearson's *χ* ^2^ test. Comparison of concentrations were conducted using Mann–Whitney nonparametric *U* test, with box and whisker plots representing the median, IQR, and range. All *p* values are numerically displayed, with a *p* < .05 considered statistically significant. Human data analyses utilized R software (version 4.0.2).

## 3 RESULTS

### 3.1 Serum G‐CSF levels are elevated in Lyn‐deficient mice

Previous studies have shown that G‐CSF levels are elevated in Lyn^−/−^IL‐10^−/−^ mice and in mice lacking Lyn in DCs.^17^, ^18^ To clarify if levels were elevated in aged Lyn^−/−^ mice, we measured serum G‐CSF, finding that levels were significantly higher in 36‐week‐old mice, compared with healthy C57BL/6 mice (Figure S1A).

### 3.2 Lyn‐deficient mice lacking G‐CSF maintain hallmarks of systemic inflammation

To determine whether G‐CSF contributes to the pathogenesis of lupus in Lyn^−/−^ mice, we generated Lyn^−/−^G‐CSF^−/−^ mice that were aged to 36 weeks and assessed alongside age‐matched C57BL/6 controls and single‐mutant Lyn^−^^/−^ and G‐CSF^−/−^ mice for hallmarks of inflammation and pathology. RT‐PCR of lung tissue confirmed that expression of the G‐CSF gene, *Csf3*, was not detected in Lyn^−/−^G‐CSF^−/−^ mice (Figure S1B). When compared with Lyn^−/−^ mice, Lyn^−/−^G‐CSF^−/−^ mice exhibited similar splenomegaly (Figure S1C), loss of splenic cellularity (Figure S1D), and splenic erythropoiesis, as indicated by the expansion of splenic erythroblasts (Ter119^+^CD71^±^) (Figure S1E,F), indicating that G‐CSF does not grossly influence inflammation in Lyn^−/−^ mice.

### 3.3 Neutrophil expansion, but not activation, is moderated in Lyn^−/−^G‐CSF^−/−^ mice

**Figure 1:**
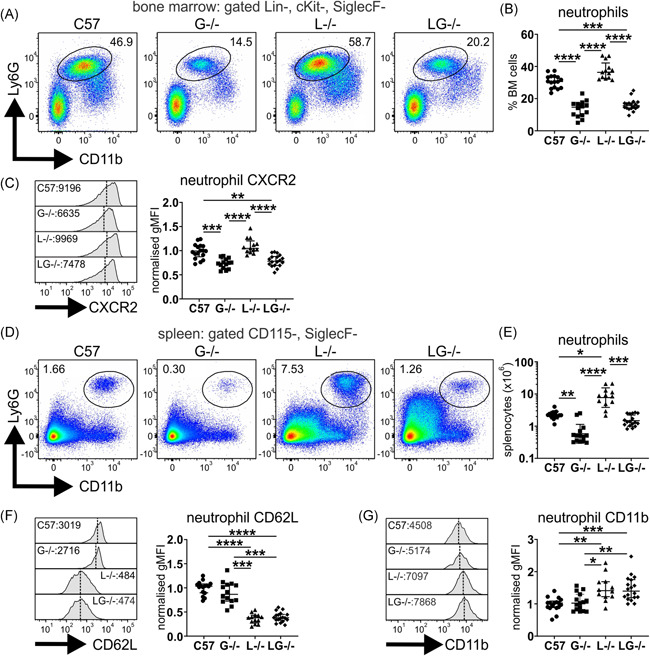
Neutrophil expansion, but not activation, is moderated in Lyn^−/−^G‐CSF^−/−^ mice. The indicated 36‐week‐old mice were evaluated by flow cytometry of BM for assessment of (A) CD11b^+^Ly6G^+^ BM neutrophils gating on Lin^−^cKit^−^SiglecF− cells; (B) quantitation of BM neutrophils in (A); (C) assessment of BM neutrophil CXCR2 expression. Flow cytometry of the spleen of 36‐week‐old mice was used to evaluate (D) CD11b^+^Ly6G^+^ spleen neutrophils gating on CD115^‐^SiglecF^‐^ cells; (E) quantitation of neutrophils in spleen from gating in (D); (F) assessment of CD62L expression on spleen neutrophils; and (G) assessment of CD11b expression on spleen neutrophils. For A, C, D, F, and G, flow cytometry plots and histograms are representative; all other data are from five experiments and *n* = 12–18 mice/genotype, with *p < .05, **p < .01, ***p < .001, ****p < .0001 by Kruskal–Wallis/Dunn's multiple comparisons test. BM, bone marrow; G‐CSF, granulocyte colony‐stimulating factor

We next assessed the impact of G‐CSF deficiency on neutrophil development in Lyn^−/−^ mice. In line with previous studies,^24^ mice deficient in G‐CSF exhibited impaired BM neutrophil numbers, which were similarly reduced in Lyn^−/−^G‐CSF^−/−^ mice (Figure 1A,B). In addition, the expression of the neutrophil maturation marker CXCR2 was similarly reduced on BM neutrophils from both G‐CSF^−/−^ and Lyn^−/−^G‐CSF^−/−^ mice (Figure 1C), indicating that G‐CSF‐deficiency is dominant in the BM.

In spleen, neutrophil numbers were largely normalized in aged Lyn^−/−^G‐CSF^−^^/−^ mice, being similar to those of C57BL/6 mice and augmented above the neutropenia of G‐CSF^−/−^ mice; however, they remained significantly below the neutrophil expansion observed in aged Lyn^−/−^ mice (Figure 1D,E). Interestingly, splenic neutrophils from aged Lyn^−/−^G‐CSF^−/−^ mice exhibited a striking loss of CD62L expression (Figure 1F) and modest upregulation of CD11b (Figure 1G), indicative of activation and in line with that in Lyn^−/−^ mice. However, this trait appears to be intrinsic to Lyn‐deficiency, as it was observed in young mice before disease onset (Figure S2). Thus, despite moderating neutrophil expansion, G‐CSF‐deficiency did not impact neutrophil activation in the periphery of Lyn^−/−^ mice.

### 3.4 G‐CSF deficiency confers minor moderation of monocyte expansion in Lyn^−/−^ mice

**Figure 2:**
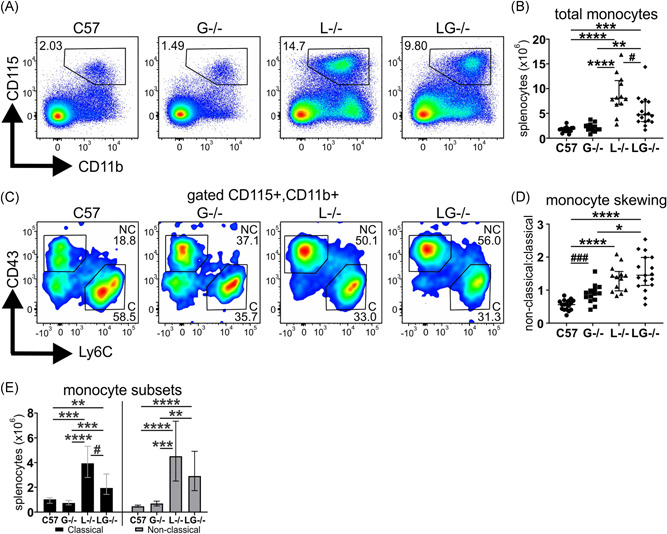
Subtle changes to the monocyte compartment of Lyn^−/−^G‐CSF^−/−^ mice. The indicated 36‐week‐old mice were evaluated by flow cytometry of spleen for assessment of (A) CD115^+^CD11b^+^ monocytes; (B) quantitation of monocyte numbers from staining in (A); (C) Ly6C^+^CD43^−^ classical and Ly6C^−^CD43^+^ nonclassical monocytes gating on CD115^+^CD11b^+^ cells; (D) ratio of nonclassical to classical monocytes from numbers derived from staining in (C); and (E) quantitation of classical and nonclassical monocyte numbers from staining in (C). For A and C, flow cytometry plots are representative; for B, D, and E, data are from five experiments and *n* = 12–18 mice/genotype, with **p < .01, ***p < .001, ****p < .0001 by Kruskal–Wallis/Dunn's multiple comparisons test and ^#^ *p* < .05, ^###^ *p* < .001 by Mann–Whitney test. G‐CSF, granulocyte colony‐stimulating factor

As G‐CSF can also act on monocytes, this compartment was examined. As reported, aged Lyn^−/−^ mice exhibited an expansion of monocytes^15^, ^19^; however, this was significantly, albeit partially, moderated in Lyn^−/−^G‐CSF^−/−^ mice (Figure 2A,B). A notable shift in monocyte polarization was observed in both Lyn^−/−^ and Lyn^−/−^G‐CSF^−/−^ mice away from homeostatic classical monocytes (Ly6C^+^CD43^−^), which were more abundant in aged C57BL/6 mice, toward inflammatory nonclassical monocytes (Ly6C^−^CD43^+^) (Figure 2C,D), with expansion of both subsets observed in Lyn^−/−^ and Lyn^−/−^G‐CSF^−/−^ mice (Figure 2E). Interestingly, this inflammatory shift was also present, but to a lesser degree, in G‐CSF^−/−^ mice. Collectively, these data indicate that deficiency of G‐CSF has a minor impact on monocyte expansion, but it does not attenuate monocyte polarization in Lyn^−/−^ mice.

### 3.5 G‐CSF does not influence the B cell and plasma cell compartments of Lyn^−/−^ mice

**Figure 3:**
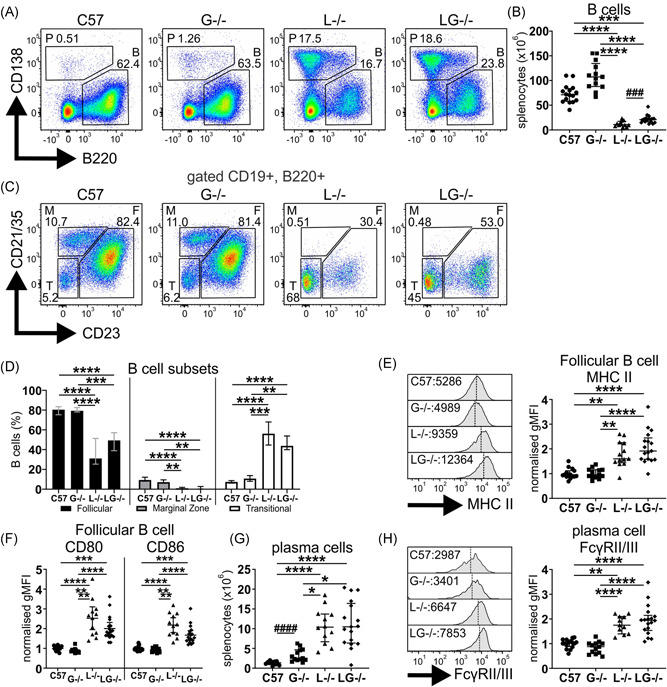
G‐CSF deficiency does not alter the B cell compartment of Lyn^−/−^ mice. The indicated 36‐week‐old mice were evaluated by flow cytometry of spleen for assessment of (A) plasma cells [P] and B cells [B] by B220 versus CD138 staining; (B) quantitation of B220^+^CD19^+^ B cells; (C) assessment of follicular [F], marginal zone [M], and transitional [T] B cells by CD23 vs CD21 staining; (D) quantitation of indicated B cell subsets from staining in (C); (E) assessment of MHCII expression on follicular B cells; (F) assessment of CD80 and CD86 expression on follicular B cells; (G) quantitation of B220^lo^CD138^+^ plasma cells from staining in (A); and (H) assessment of FcγRII/III expression on plasma cells. For A, C, E, and H, flow cytometry plots and histograms are representative; for B and D–H, data are from 5 experiments and *n* = 12–18 mice/genotype, with *p < .05, **p < .01, ***p < .001, ****p < .0001 by Kruskal–Wallis/Dunn's multiple comparisons test and ^###^ *p* < .001 by Mann–Whitney test. G‐CSF, granulocyte colony‐stimulating factor

Lyn^−/−^ mice exhibit B cell developmental defects and show B cell hyperactivation, which facilitates their autoimmune pathology, and thus the impact of G‐CSF‐deficiency on this compartment was examined. Aged Lyn^−/−^ mice exhibited typical B cell lymphopenia (CD19^+^B220^+^), which persisted in Lyn^−/−^G‐CSF^−/−^ mice; however, numbers of B cells were slightly recovered (Figure 3A,B). In addition, both Lyn^−/−^ and Lyn^−/−^G‐CSF^−/−^ mice showed reduced follicular B cells (CD21/35^lo^CD23^+^), complete loss of marginal zone B cells (CD21/35^hi^CD23^−^), and expansion of the transitional B cell compartment (CD21/35^−^CD23^−^) (Figure 3C,D). Similarly, follicular B cells of Lyn^−/−^G‐CSF^−/−^ mice exhibited enhanced expression of MHCII (Figure 3E) as well as CD80 and CD86 (Figure 3F), as observed in Lyn^−/−^ mice. Whereas G‐CSF‐deficient mice displayed a minor expansion of plasma cells, marked plasmacytosis was equivalently observed in both Lyn^−/−^ and Lyn^−/−^G‐CSF^−/−^ mice (Figures 3A and 3G), with plasma cells from both genotypes exhibiting similarly augmented expression of the immunoregulatory FcγRII/III (Figure 3H). Overall, this indicates that G‐CSF is not implicated in the developmental defects or hyperactivation of the B cell and plasma cell compartments in Lyn^−/−^ mice.

### 3.6 G‐CSF does not alter the DC phenotype of Lyn^−/−^ mice

DCs are strongly associated with the lupus disease process in Lyn^−/−^ mice^18^ and in human SLE,^25^ and G‐CSF is implicated in modulation of DC function and the promotion of anergic T cells.^26^ Phenotyping of conventional DCs (cDCs) of aged mice revealed that both Lyn^−/−^ and Lyn^−/−^G‐CSF^−/−^ mice exhibited a significant deficit in the splenic cDC1 subset (CD11c^hi^MHCII^+^CD8α^+^CD172α^−^), whereas the cDC2 subset (CD11c^hi^MHCII^+^CD8α^‐^CD172α^+^) was unaffected (Figure S3A–C). On further examination, expression levels of CD86 were normal on cDC1, but elevated on cDC2 of both Lyn^−/−^ and Lyn^−/−^G‐CSF^−/−^ mice (Figure S3D and Figure 3E), indicative of a hypermature phenotype. These data suggest that G‐CSF deficiency does not alter the cDC phenotype of Lyn^−/−^ mice.

### 3.7 CD4^+^ effector memory T cells are expanded in Lyn^−/−^G‐CSF^−^^/−^ mice

**Figure 4:**
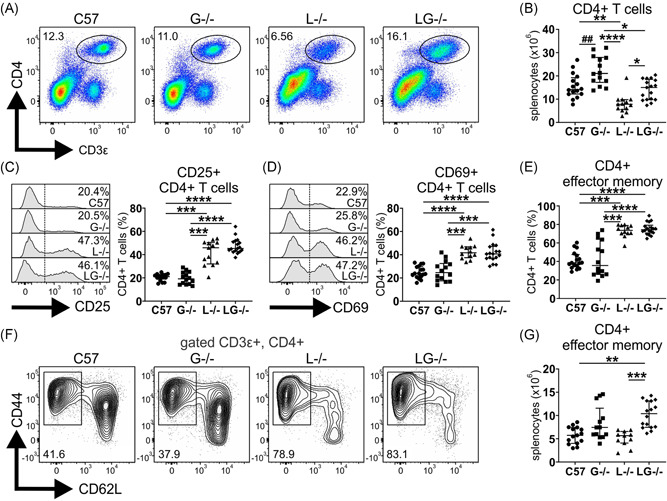
CD4^+^ effector memory T cells are expanded in Lyn^−/−^G‐CSF^−/−^ mice. The indicated 36‐week‐old mice were evaluated by flow cytometry of spleen for assessment of (A) CD3ε^+^CD4^+^ T cells; (B) quantitation of CD3ε^+^CD4^+^ T cells from staining in (A); (C) assessment and quantitation of CD25^+^CD4^+^ T cells; (D) assessment and quantitation of CD69^+^CD4^+^ T cells; (E) proportions of effector memory (CD44^+^CD62L^−^) CD4^+^ T cells from staining in (F); (F) assessment of CD62L vs CD44 on gated CD3ε^+^CD4^+^ T cells; and (G) quantitation of CD44^+^CD62L^−^ effector memory T cells from staining in (F). For A, C, D, and F, flow cytometry plots and histograms are representative; for B, C, D, E, and G, data are from five experiments and *n* = 12–18 mice/genotype, with *p < .05, **p < .01, ***p < .001, ****p < .0001 by Kruskal–Wallis/Dunn's multiple comparisons test and ^##^ *p* < .01 by Mann–Whitney test

T cells are implicated in the pathogenesis of autoimmune disease in Lyn^−/−^ mice, where they display inflammation‐driven hyperactivation,^15^, ^19^, ^27^ and, therefore, we examined whether G‐CSF influenced this phenotype. Unexpectedly, aged G‐CSF^−/−^ mice exhibited an expansion of CD4^+^ T cells, and contrary to the well‐established CD4^+^ T cell lymphopenia observed in aged Lyn^−/−^ mice, Lyn^−/−^G‐CSF^−/−^ mice had normal numbers of CD4^+^ T cells, comparable to C57BL/6 controls (Figure 4A,B). CD4^+^ T cells from both Lyn^−/−^ and Lyn^−/−^G‐CSF^−/−^ displayed a similarly activated phenotype with greater proportions of cells expressing the activation markers CD25 (Figure 4C) and CD69 (Figure 4D). Although phenotypic subsetting revealed that the CD4^+^ T cells of both Lyn^−/−^ and Lyn^−/−^G‐CSF^−/−^ mice were skewed toward an effector memory (CD44^+^CD62L^−^) phenotype (Figure 4E,F), an expansion of numbers was only seen in Lyn^−/−^G‐CSF^−/−^ mice due to their increase in total CD4^+^ T cells, compared with Lyn^−/−^ mice (Figure 4G).

Examination of CD8^+^ T cells revealed no differences in CD8^+^ T cell lymphopenia, skewing to an effector memory phenotype (CD44^+^CD62L^−^) and enhanced CD69 expression between Lyn^−/−^ and Lyn^−/−^G‐CSF^−/−^ mice (Figure S4A–D).

### 3.8 G‐CSF deficiency exacerbates autoimmunity and kidney pathology in Lyn^−/−^ mice

**Figure 5:**
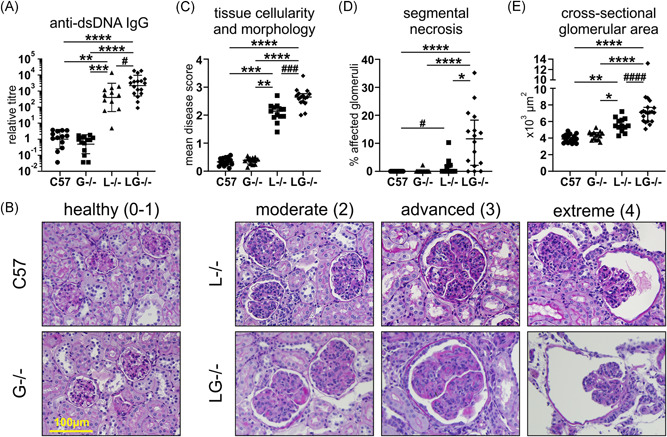
G‐CSF deficiency augments autoantibodies and kidney pathology in Lyn^−/−^ mice. The indicated 36‐week‐old mice were evaluated for (A) anti‐dsDNA IgG autoantibodies; and (B) kidney pathology, showing examples of healthy glomeruli of C57BL/6 and G‐CSF^−/−^ mice and moderate, advanced, and extreme disease in Lyn^−^^/−^ and Lyn^−/−^G‐CSF^−^^/−^ mice; (C) mean disease score of glomerular hypercellularity and dysregulation of tissue morphology; (D) frequency of glomeruli exhibiting necrotic lesion; and (E) quantitation of mean glomerular cross‐sectional area. For A and C–E, data are from *n* = 12–18 mice/genotype, and for C–E, each data point represents a single mouse with ≥30 glomeruli/mouse assessed. *p < .05, **p < .01, ***p < .001, ****p < .0001 by Dunn's multiple comparisons test and ^#^ *p* < .05, ^###^ *p* < .001, ^####^ *p* < .0001 by Mann–Whitney test. dsDNA, double stranded DNA; G‐CSF, granulocyte colony‐stimulating factor; IgG, immunoglobulin G

We next assessed the contribution of G‐CSF to autoimmunity and kidney pathology in Lyn‐deficient mice. Autoantibody titers were minimal in aged G‐CSF^−/−^ mice, comparable to those of C57BL/6 mice (Figure 5A). Although variable, Lyn^−/−^ mice developed, on average, autoantibody titers that were 398 times greater than those detected in C57BL/6 mice, which were further augmented in Lyn^−/−^G‐CSF^−/−^ mice, being five times greater than titers in Lyn^−/−^ mice (Figure 5A). Kidney pathology was assessed via three independent histopathological measures: scoring of glomerular hypercellularity and dysregulation of tissue morphology; assessing frequency of glomeruli exhibiting necrotic lesions (segmental necrosis); and measuring glomerular expansion (cross‐sectional area). These histopathological studies showed that kidneys from aged G‐CSF^−/−^ mice were healthy with glomeruli exhibiting a normal rounded morphology with only mild subcapsular space enlargement, no indication of cellular infiltration (median tissue score 0.39, IQR: 0.26–0.46) with negligible segmental necrosis, and standard cross‐sectional area (median area 4396 μm^2^, IQR: 3801–4335), comparable to aged‐matched C57BL/6 mice (median tissue score 0.33, IQR: 0.25–0.46; median area 3888 μm^2^, IQR: 3451–4666) (Figure 5B–E). Conversely, both aged Lyn^−/−^ mice and Lyn^−/−^G‐CSF^−/−^ mice exhibited varying degrees of glomerular injury, denoted as moderate, advanced, and extreme, which correlate with the described scoring criteria of 0–4 (Figure 5B). Lyn^−/−^G‐CSF^−/−^ mice exhibited further glomerular hypercellularity and dysregulation of tissue morphology (median tissue score 2.65, IQR: 2.47–2.77) with markedly increased occurrence of segmental necrosis (median necrosis freq. 11.62%, IQR: 2.15–18.28) and enhanced glomerular expansion (median area 7106 μm^2^, IQR: 6035–7704), compared with Lyn^−/−^ mice (median tissue score 2.14, IQR: 1.94–2.30; median necrosis freq. 0.0%, IQR: 0.00–2.14; median area 5471 μm^2^, IQR: 5079–6247) (Figure 5B–E). Collectively, these data suggest that deficiency of G‐CSF augments the development of autoimmunity and resultant kidney pathology in Lyn^−/−^ mice.

### 3.9 Serum G‐CSF levels are not increased in patients with lupus or those with active renal disease

**Figure 6:**
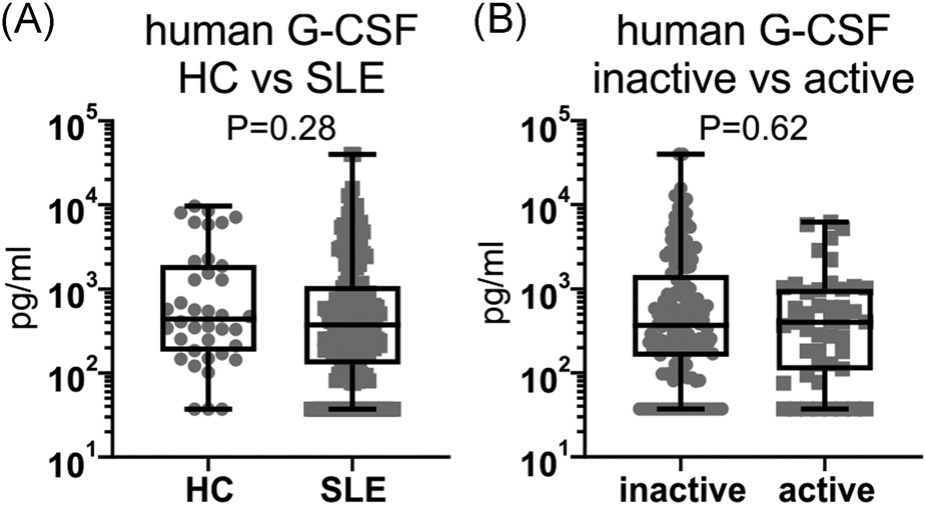
Serum G‐CSF does not correlate with human lupus or with renal manifestations of lupus. (A) Serum G‐CSF concentrations in human healthy controls (HC, *n* = 38) and SLE patients (SLE, *n* = 198). (B) Serum G‐CSF concentrations in human SLE patients with inactive (*n* = 152) and active (*n* = 46) renal disease. The *p* values were derived using Mann–Whitney test. G‐CSF, granulocyte colony‐stimulating factor; HC, healthy control; SLE, systemic lupus erythematosus

To determine whether G‐CSF is implicated in human disease, G‐CSF was measured in the serum of 198 SLE patients and 38 healthy controls (HCs) (Table S1). G‐CSF was detectable in 84.3% (167/198) of SLE patients and 92.1% (35/38) of HC (*p* = .21, Pearson's *χ* ^2^ test), and there was no significant difference in serum G‐CSF concentrations between patients with SLE (median: 377.1 pg/ml, IQR: 133.6–1067.2) and HC (median: 440.5 pg/ml, IQR: 192.2–1799.2) (Figure 6A). Furthermore, there was no difference in the proportion of SLE patients with detectable serum G‐CSF concentrations between SLE patients with active (82.6%, 38/46) or inactive (84.9%, 129/152) renal disease (*p* = .71, Pearson's *χ* ^2^ test), nor in the serum G‐CSF concentrations between SLE patients with active (median: 404.0 pg/ml, IQR: 115.4–990.8) or inactive (median: 371.4 pg/ml, IQR: 161.5–1350.2) renal disease (Figure 6B). This indicates that serum G‐CSF levels do not correlate with lupus incidence or kidney disease activity in humans.

## 4 DISCUSSION

In this study, we have assessed whether the myeloid growth factor G‐CSF contributes to disease pathogenesis in Lyn‐deficient mice, which develop severe lupus nephritis over time, as we found that levels of G‐CSF are elevated in the circulation of aged mice. Previously, Lyn^−/−^ mice have also been shown to exhibit expansion of myeloid cells as disease ensues,^15^, ^19^ which may collectively be a consequence of loss of negative regulation of signaling from the G‐CSF receptor^28^ and elevated systemic G‐CSF levels. Furthermore, Lyn‐deficient neutrophils have a hyperadhesive and hyperactive phenotype,^20^ suggesting that they contribute to disease. G‐CSF is known to induce upregulation of CD11b and downregulation of CD62L on neutrophils,^29^ which are features of Lyn‐deficient neutrophils, hinting that elevated G‐CSF could drive neutrophil activation. However, in our study, while neutrophil numbers in Lyn^−/−^ mice were impacted by G‐CSF deficiency, they maintained their activated phenotype. Given that this phenotype is also observed in young mice, which precedes onset of kidney pathology, it is likely that it is intrinsic to Lyn deficiency in these cells. Lyn^−/−^ mice also display extramedullary splenic hematopoiesis that occurs in response to inflammation and disease, and although G‐CSF is known to promote blood stem cell production and mobilization,^21^ our data implicate other growth factors in this process, in addition to G‐CSF, as myeloid cell development and expansion were only partially moderated in Lyn^−/−^G‐CSF^−/−^ mice.

Our analysis of aged mice, when Lyn^−/−^ mice normally exhibit severe lupus nephritis, indicates that deficiency of G‐CSF did not attenuate disease. Although Lyn^−/−^G‐CSF^−/−^ mice exhibited similar B cell compartmental defects ordinarily detected in Lyn^−/−^ mice, they had enhanced autoantibody production, which was almost certainly driven by their augmented expansion of effector CD4^+^ T cells. G‐CSF has been described as a repressor of T cell proliferation, and it can promote immunoregulatory functions in T cells such as IL‐10 production,^30^, ^31^ so these protective features may be lost in Lyn^−/−^G‐CSF^−/−^ mice. G‐CSF administration has been shown to promote the generation of regulatory DCs that are impaired in IL‐12 production and T cell stimulation,^26^, ^32^ so this may be another feature that is altered in Lyn^−/−^G‐CSF^−/−^ mice. However, we did not observe any phenotypic changes in cDC maturation or activation beyond those already observed in Lyn^−/−^ mice.

Furthermore, we found that kidney pathology was also exacerbated in Lyn^−/−^G‐CSF^−/−^ mice. Previously, lupus‐prone MRL‐*lpr* mice treated with a low dose of G‐CSF exhibited exacerbated kidney disease with enhanced glomerular deposition of Ig, whereas those on high dose treatment were protected, possibly by local downmodulation of Fc receptor expression in the glomeruli.^33^ Similarly, in the NZB/W mouse model of lupus, daily treatment with G‐CSF from disease onset for 12 weeks restrained lupus nephritis, attenuated leukocyte infiltration into kidney, promoted regulatory T cell expansion, and reduced proinflammatory cytokines.^34^ Together with our results, this demonstrates that the effects of G‐CSF on disease development are complex and maybe dose and disease stage‐dependent. The increase in serum G‐CSF levels may be an important compensatory mechanism, with its loss in Lyn^−/−^G‐CSF^−/−^ mice resulting in a more severe disease presentation. However, it is also important to consider that some lupus patients exhibit neutropenia,^35^ which has occasionally been treated with recombinant human G‐CSF.^36^ Although this can rapidly augment neutrophil numbers, there have been reports of disease flares in some patients,^37^, ^38^, ^39^ indicating that G‐CSF may have deleterious effects in certain SLE settings. This may be determined by the specific disease‐associated genetic mutations present. For example, SHIP‐1‐deficient mice represent another lupus model with systemic inflammation, elevated G‐CSF levels, enhanced extramedullary hematopoiesis, and myeloid cell expansion.^40^ Although many of these features are dependent on the presence of G‐CSF, loss of G‐CSF did not rescue autoimmunity, as SHIP‐1^−/−^G‐CSF^−/−^ mice exhibited activated B cells, elevated Ig levels, circulating autoantibodies, and glomerulonephritis.^7^ Also, the lupus‐prone B6.*Sle1.2.3* triple‐congenic mouse represents a complex model that harbors a number of interacting genes that impart susceptibility or resistance to lupus. One region is *Sle2c* that encodes several genes including the *G‐CSF receptor* (*Csf3r*), which carries a nonsynonymous mutation in the extracellular domain that confers resistance to lupus.^41^, ^42^ This is a loss‐of‐function mutation, as G‐CSF binding was impaired and G‐CSF treatment failed to mobilize BM neutrophils, indicating that B6.*Sle2c2* mice are resistant to developing lupus due to their inability to mobilize G‐CSF‐responsive BM cells.^43^ These collective studies indicate that there is heterogeneity in lupus across different disease models, with some reliant on G‐CSF signaling, some protected by exogenous G‐CSF treatment, and others seemingly independent of this growth factor. Elevated G‐CSF has been found in the cerebrospinal fluid but not the serum of SLE patients with neuropsychiatric lupus, compared with those without this disease trait^44^; however, in our human cohort, there was an overall lack of correlation between serum G‐CSF levels and the incidence of lupus as well as the renal disease activity of patients. This suggests that serum G‐CSF is a poor biomarker of systemic disease and is unlikely to be a major influential factor in the disease processes involved in human lupus nephritis.

In summary, we have examined if G‐CSF‐dependent inflammation participates in lupus. We have shown that Lyn^−/−^G‐CSF^−/−^ mice have reduced tissue and BM neutrophils, but they retain other immune attributes of Lyn‐deficient mice such as augmented neutrophil activation and B cell compartment defects. However, Lyn^−/−^G‐CSF^−/−^ mice exhibit more profound T cell activation, develop increased autoantibody titers, and display more severe glomerulonephritis with age, compared with Lyn^−/−^ mice. These studies show that G‐CSF is nonpathogenic and, in fact, may play a protective role in this experimental lupus model. In addition, we found that serum G‐CSF levels did not correlate with lupus in humans. Overall, our studies do not support the use of G‐CSF antagonist‐targeted therapies to treat this inflammatory autoimmune disease.

## CONFLICT OF INTERESTS

The authors declare that there are no conflicts of interests.

## Supporting information

Supporting information.

## Data Availability

Data are available on request due to privacy/ethical restrictions.
