# Supplementary material for: Granulocyte colony‐stimulating factor is not pathogenic in lupus nephritis
Source: Immun Inflamm Dis. 2021 May 7;9(3):758–70. doi: 10.1002/iid3.430 (PMC8342225; doi:10.1002/iid3.430)
Supplement: Supplementary file 1 — Supporting information. [file IID3-9-758-s001.pdf]

S1

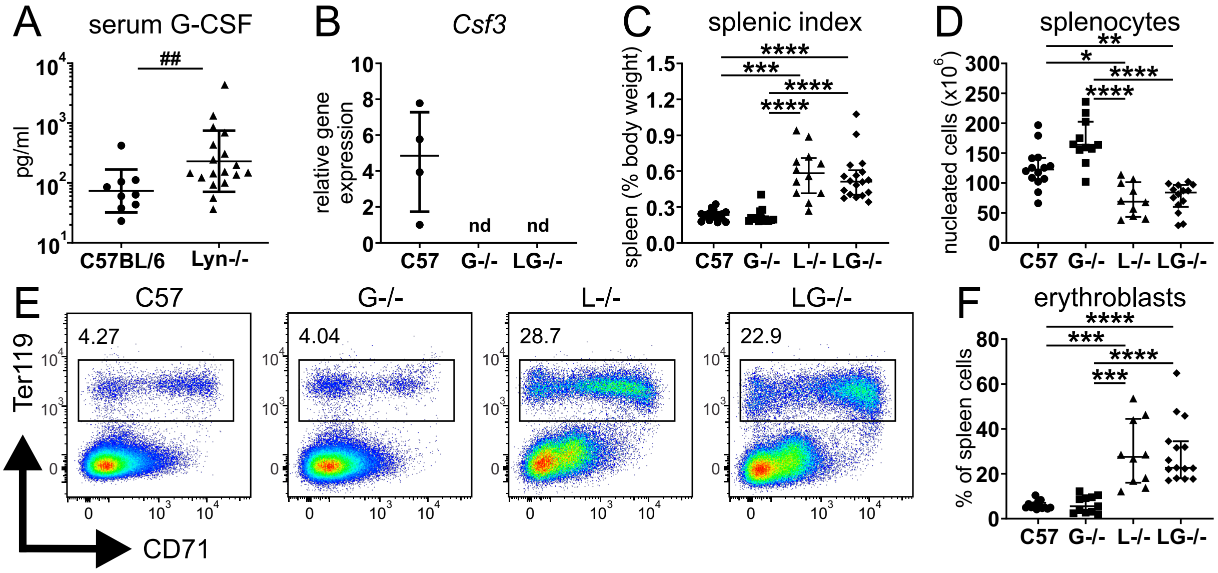

Supplemental Figure 1. G-CSF levels are elevated in aged Lyn$^{-/-}$ mice but loss of G-CSF does not influence gross inflammatory features. (A) G-CSF levels in the serum of 36-wk-old C57BL/6 (n=9) and Lyn$^{-/-}$ mice (n=18). (B) Relative expression of the *Csf3* gene in lung tissue of C57BL/6 (n=4), G-CSF$^{-/-}$ (n=5) and Lyn$^{-/-}$G-CSF$^{-/-}$ (n=4) mice, nd = not detected. The indicated 36-week-old mice were assessed for (C) splenic index (spleen weight as a proportion of body weight); (D) total number of nucleated splenocytes; (E) representative flow cytometry staining for erythroblasts; and, (F) proportions of Ter119^{+}CD71$^{\pm}$ erythroblasts per spleen from staining in (D). For A, ## $p < 0.01$ by Mann-Whitney test. For C, D and F, data are from 4-5 experiments and n=10-18 mice/genotype, with * $p < 0.05$, ** $p < 0.01$, *** $p < 0.001$, **** $p < 0.0001$ by Kruskal-Wallis/Dunn's multiple comparisons test.

S2

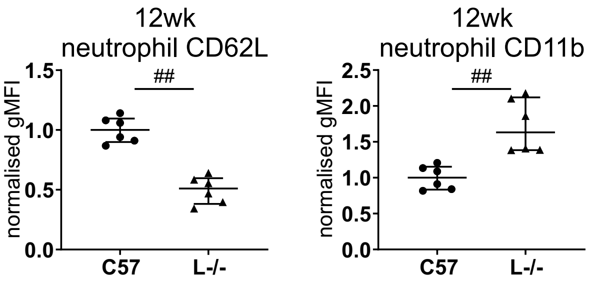

Supplemental Figure 2. Lyn$^{-/-}$ mice display aberrant neutrophil activation from a young, pre-disease age. The indicated 12-week-old mice were evaluated by flow cytometry of splenic neutrophils for expression of CD62L and CD11b. Data are from 2 experiments and n=6 mice/genotype, with ## $p < 0.01$ by Mann-Whitney test.

S3

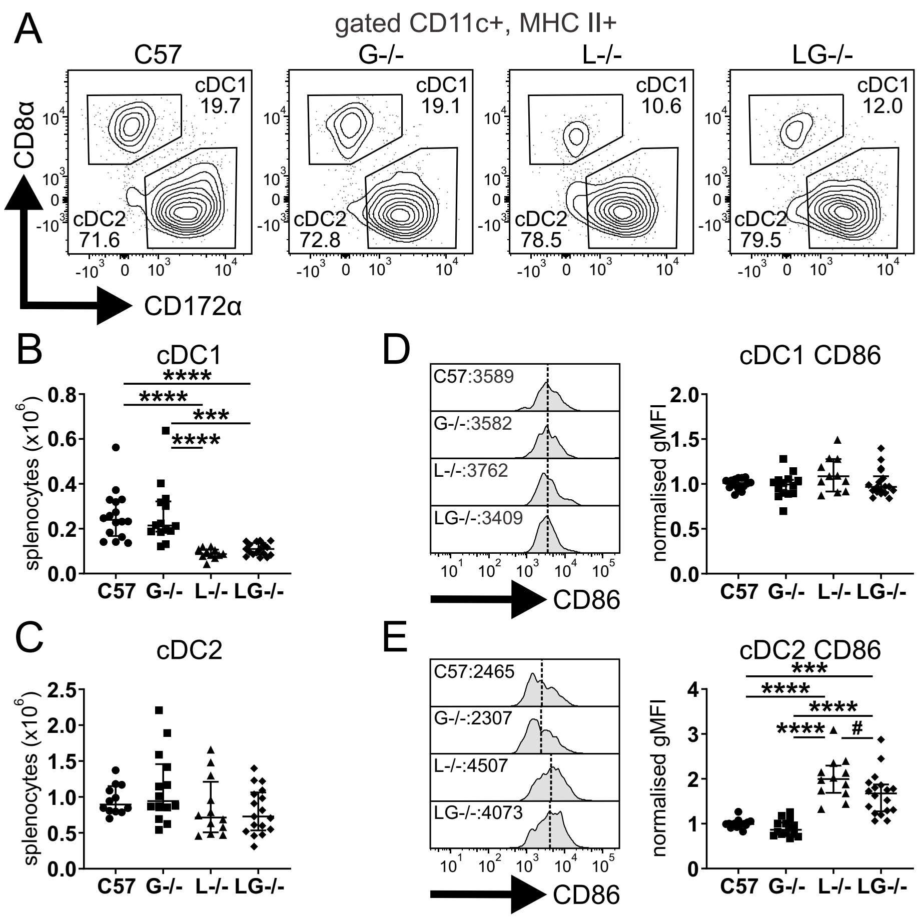

Supplemental Figure 3. G-CSF deficiency does not influence cDCs of Lyn$^{-/-}$ mice. The indicated 36-week-old mice were evaluated by flow cytometry of spleen for assessment of (A) cDC1 (CD8α^{+}) and cDC2 (CD172α^{+}) DC subsets pre-gated on CD11c^{+}MHCII^{+}; and quantitation of (B) CD8α^{+}CD172α^{-} cDC1 from staining in (A); (C) CD8α^{-}CD172α^{+} cDC2 from staining in (A); (D) CD86 expression on cDC1; (E) CD86 expression on cDC2. For A, D and E, flow cytometry plots and histograms are representative; for B, C, D and E, data are from 5 experiments and n=12-18 mice/genotype, with *** $p < 0.001$, **** $p < 0.0001$ by Kruskal-Wallis/Dunn's multiple comparisons test and # $p < 0.01$ by Mann-Whitney test.

S4

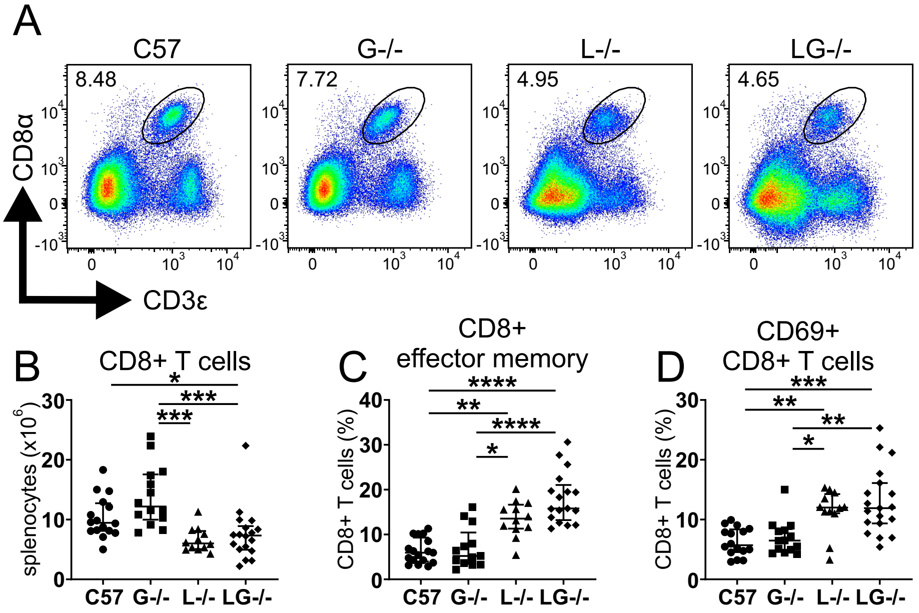

Supplemental Figure 4. G-CSF deficiency does not alter the $CD8^+$ T cell compartment of Lyn-deficient mice. The indicated 36-week-old mice were evaluated by flow cytometry of spleen for assessment of (A) $CD3\varepsilon^+CD8^+$ T cells (representative flow plots); and (B) quantitation of $CD3\varepsilon^+CD8^+$ T cells; (C) $CD44^+CD62L^-$ effector memory $CD8^+$ T cells; and, (D) activated $CD69^+CD3\varepsilon^+CD8^+$ T cells. For B-D, data are from 5 experiments and n=12-18 mice/genotype, with * $p < 0.05$, ** $p < 0.01$, *** $p < 0.001$, **** $p < 0.0001$ by Kruskal-Wallis/Dunn's multiple comparisons test.

Supplemental Table 1. Characteristics of human study participants

	SLE patients (SLE)	Healthy control (HC)
**Number of participants**	198	38
**Female, n (%)**	175 (88.4%)	33 (86.8%)
**Age in yrs, median (IQR)**	42.4 (32.7 – 52.1)	37.6 (30.8 – 46.2)
**Asian ethnicity, n (%)**	92 (46.5%)	16 (42.1%)
**Disease duration in yrs, median (IQR)**	8.1 (3.8 – 16)	N/A
**Active renal disease, n (%)**	46 (23.2%)	N/A
